# Cooperative Action of Cdk1/cyclin B and SIRT1 Is Required for Mitotic Repression of rRNA Synthesis

**DOI:** 10.1371/journal.pgen.1005246

**Published:** 2015-05-29

**Authors:** Renate Voit, Jeanette Seiler, Ingrid Grummt

**Affiliations:** Division of Molecular Biology of the Cell II, German Cancer Research Center, DKFZ-ZMBH Alliance, Heidelberg, Germany; J. David Gladstone Institutes, and the University of California, San Francisco, UNITED STATES

## Abstract

Mitotic repression of rRNA synthesis requires inactivation of the RNA polymerase I (Pol I)-specific transcription factor SL1 by Cdk1/cyclin B-dependent phosphorylation of TAF_I_110 (TBP-associated factor 110) at a single threonine residue (T852). Upon exit from mitosis, T852 is dephosphorylated by Cdc14B, which is sequestered in nucleoli during interphase and is activated upon release from nucleoli at prometaphase. Mitotic repression of Pol I transcription correlates with transient nucleolar enrichment of the NAD^+^-dependent deacetylase SIRT1, which deacetylates another subunit of SL1, TAF_I_68. Hypoacetylation of TAF_I_68 destabilizes SL1 binding to the rDNA promoter, thereby impairing transcription complex assembly. Inhibition of SIRT1 activity alleviates mitotic repression of Pol I transcription if phosphorylation of TAF_I_110 is prevented. The results demonstrate that reversible phosphorylation of TAF_I_110 and acetylation of TAF_I_68 are key modifications that regulate SL1 activity and mediate fluctuations of pre-rRNA synthesis during cell cycle progression.

## Author Summary

In metazoans, transcription is arrested during mitosis. Previous studies have established that mitotic repression of cellular transcription is mediated by Cdk1/cyclin B-dependent phosphorylation of basal transcription factors that nucleate transcription complex formation. Repression of rDNA transcription at the onset of mitosis is brought about by inactivation of the TBP-containing transcription factor SL1 by Cdk1/cyclin B-dependent phosphorylation of the TAF_I_110 subunit, which impairs the interaction with UBF and the assembly of pre-initiation complexes. Here we show that hCdc14B, the phosphatase that regulates Cdk1/cyclin B activity and progression through mitosis, promotes reactivation of rDNA transcription by dephosphorylating TAF_I_110. In addition, the NAD^+^-dependent deacetylase SIRT1 becomes transiently enriched in nucleoli at the onset of mitosis. SIRT1 deacetylates TAF_I_68, another subunit of SL1, hypoacetylation of TAF_I_68 destabilizing SL1 binding to the rDNA promoter and impairing transcription complex assembly. The results reveal that modulation of SL1 activity by reversible acetylation of TAF_I_68 and phosphorylation of TAF_I_110 are key modifications that mediate oscillation of rDNA transcription during cell cycle progression.

## Introduction

Posttranslational modification of transcription factors is critical for cell cycle progression in a unidirectional and reversible manner. Cell cycle-dependent oscillation of transcriptional activity is governed by a complex network of regulatory proteins and signaling pathways that respond to various intra- and extracellular stimuli by influencing the activity and tertiary structure of proteins, controlling subcellular distribution, and regulating interactions with other proteins. Global repression of gene expression starts at prophase and is accompanied by release of most transcriptional regulators from mitotic chromatin [1–3, 4]. Mitotic switch-off of cellular transcription involves inactivation of key components of the transcription machinery. For class II genes, components of the basal transcription apparatus are inactivated by mitotic phosphorylation, including TAF subunits of TFIID [4, 5], the cdk7 subunit of TFIIH [6, 7] and the heptapeptide repeats of the carboxy-terminal domain (CTD) of RNA polymerase II [8]. For class III genes, inactivation of TFIIIB causes repression of RNA polymerase III (Pol III) transcription [9–11].

With regard to transcription by RNA polymerase I (Pol I), the nucleolar structure undergoes extensive changes at the onset of mitosis, and rDNA transcription ceases between pro-metaphase and telophase [3]. While most nucleolar proteins disperse throughout the mitotic cell after breakdown of the nuclear envelope, some components of the Pol I transcription machinery, including UBF and TTF-I, remain associated with nucleolus organizer regions (NORs) to bookmark active rDNA repeats [11, 12]. Consistent with post-translational modification of basal transcription factors controlling cell cycle-dependent fluctuations of gene expression, mitotic silencing and reactivation of rDNA transcription upon mitotic exit has been shown to be governed by reversible phosphorylation of the promoter selectivity factor SL1 [13]. SL1 is a multiprotein complex comprising the TATA-box binding protein (TBP) and five TBP-associated factors (TAF_I_s), TAF_I_110, TAF_I_68, TAF_I_48, TAF_I_41, and TAF_I_12 [14–17]. At the onset of mitosis, Cdk1/cyclin B, the kinase that triggers early mitotic events, e.g. chromosome condensation, nuclear envelope breakdown and spindle pole assembly, phosphorylates TAF_I_110. This phosphorylation impairs the interaction between SL1 and UBF, thus attenuating the assembly of pre-initiation complexes at the rDNA promoter [13, 18]. Upon exit from mitosis, rDNA transcription is restored, yet the mechanisms that restore transcriptional activity are poorly characterized [19].

In this study, we have investigated the molecular mechanisms that cause reversible mitotic inactivation of SL1 at the onset of mitosis and relieve transcriptional silencing at the end of mitosis. Consistent with prior studies showing that the phosphatase hCdc14B regulates progression through mitosis by counteracting mitotic phosphorylation by Cdk1/cyclin B [20], hCdc14B dephosphorylates TAF_I_110, thus promoting its reactivation as cells exit mitosis. Notably, though phosphorylation of TAF_I_110 by Cdk1/cyclin B is necessary, alone it is not sufficient for mitotic inactivation of rDNA transcription. Previous studies have established that another SL1 subunit, TAF_I_68, is acetylated by PCAF, acetylation of TAF_I_68 stabilizing binding of SL1 to the rDNA promoter [21]. Here we show that deacetylation of TAF_I_68 by the NAD^+^-dependent deacetylase SIRT1 is also crucial for mitotic inactivation of SL1. SIRT1 becomes enriched in nucleoli at the onset of mitosis and deacetylates TAF_I_68, which in turn weakens SL1 binding to rDNA and impairs transcription complex assembly. Thus both phosphorylation of TAF_I_110 by Cdk1/cyclin B and deacetylation of TAF_I_68 by SIRT1 are required for repression of Pol I transcription during mitosis. The finding that both Cdk1 and SIRT1 modulate the activity of SL1 underscores the functional significance of reversible modification of SL1 in linking cycle progression to regulation of rDNA transcription.

## Results

### The phosphatase Cdc14B counteracts mitotic phosphorylation of TAF_I_110

**Fig 1:**
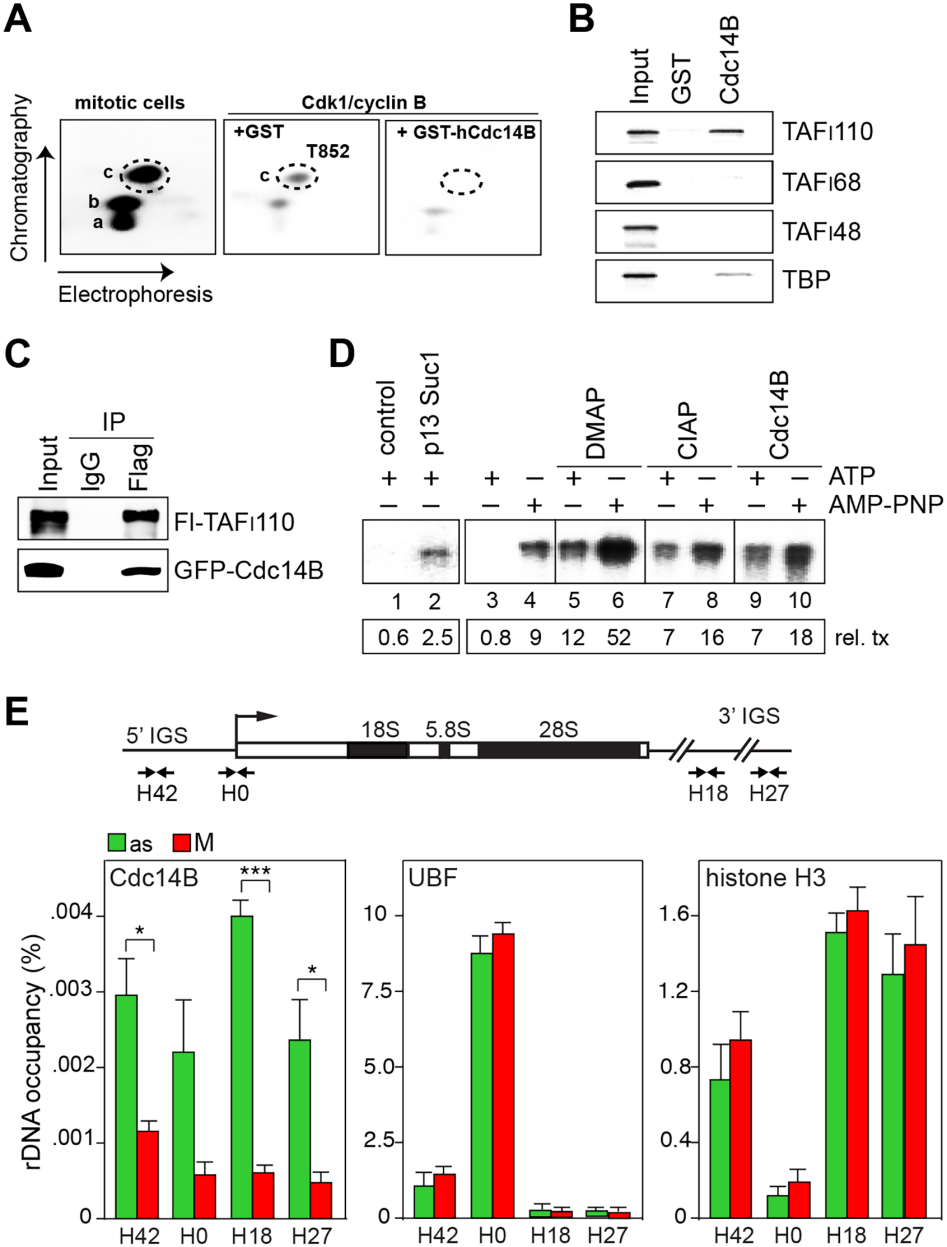
Cdc14B relieves transcriptional repression by dephosphorylating TAF_I_110 at threonine 852. (A) hTAF_I_110 is phosphorylated at T852 during mitosis. Tryptic phosphopeptide mapping of TAF_I_110. Left: Tryptic phosphopeptide map of Flag-TAF_I_110 radiolabeled with extract from mitotic HeLa cells in the presence of [^32^P]-ATP. Left: Peptide **c** (encircled) co-migrates with the synthetic phosphopeptide (SQQHpTPVLSSQPLR). Middle and right: Tryptic phosphopeptide maps of Flag-TAF_I_110 radiolabeled with purified Cdk1/cyclin B, and incubated with GST or GST-hCdc14B for 1 h at 37°C in the presence of DMAP. (B) hCdc14B interacts with TAF_I_110. GST or GST-hCdc14B were incubated for 4 h at 4°C with ^35^S-labeled hTAF_I_110, hTAF_I_68, hTAF_I_48, or hTBP. GST-bound proteins were separated by SDS-PAGE and visualized by PhosphorImaging. 10% of input proteins were loaded. (C) TAF_I_110 interacts with hCdc14B. HEK293T cells expressing Flag-hTAF_I_110 and GFP-hCdc14B were treated with nocodazole (80 ng/ml) for 23 h and released from the nocodazole arrest for 30 min. Flag-hTAF_I_110 was immunoprecipitated with M2 antibodies and co-precipitated hCdc14B was monitored on Western blots using anti-GFP antibodies. (D) hCdc14B counteracts Cdk1-mediated mitotic repression of Pol I transcription. Lanes 1, 2: Cdk1/cyclin B was depleted from mitotic extract by pre-incubation with bead-bound p13suc1 and the supernatant was assayed for transcriptional activity. Lanes 3–10: Extract from mitotic HeLa cells was assayed for transcriptional activity in the presence of ATP or AMP-PNP, DMAP, CIAP, or GST-hCdc14B as indicated. The numbers below show the relative amount of run-off transcripts. See also S1E Fig. (E) hCdc14B is released from rDNA during mitosis. The bar diagrams present ChIP data showing rDNA occupancy of Cdc14B, UBF and histone H3 in asynchronous cells (as, green bars) and nocodazole-treated mitotic U2OS cells (M, red bars). Bars denote means ±SD from three independent biological replicates (*p < 0.02; ***p < 0.001). See also S1F and S1G Fig, and S1 Table.

In asynchronous cells, TAF_I_110 (TAF1C) is constitutively phosphorylated at two tryptic peptides (labeled **a** and **b** in Fig 1A). In mitotic cells, a third peptide (labeled **c**) is phosphorylated by Cdk1/cyclin B, phosphorylation of peptide **c** correlating with mitotic inactivation of SL1 and transcriptional repression [13]. Phosphoamino acid analysis using 2-dimensional electrophoresis along with amino acid standards of phosphorylated serine, threonine and tyrosine showed that Cdk1/cyclin B phosphorylates TAF_I_110 at threonine, phosphorylation being reduced by the Cdk inhibitor roscovitine (S1A Fig). Two-dimensional phosphopeptide mapping experiments revealed that peptide **c** co-migrates with a synthetic peptide (SQQHpTPVLSSQPLR) that is phosphorylated at threonine 852 (Fig 1A and S1B Fig), suggesting that phosphorylation of T852 is causally involved in mitotic inactivation of SL1. Sequence alignment revealed that T852 as well as adjacent amino acids are conserved in TAF_I_110 from different vertebrates (S1C Fig). Regarding the phosphatase that counteracts mitotic phosphorylation of T852, we hypothesized that hCdc14B, the phosphatase that regulates Cdk1/cyclin B activity and progression through mitosis in mammals [20], could remove the inhibitory phosphate from T852. Indeed, recombinant hCdc14B dephosphorylated peptide **c** comprising T852, but not peptides **a** or **b** (Fig 1A, right). *In vitro* protein pull-down assays showed specific association of hCdc14B with hTAF_I_110 (Fig 1B). Moreover, co-immunoprecipitation experiments demonstrate that hCdc14B interacts with hTAF_I_110 *in vivo* (Fig 1C), supporting that hCdc14B is the phosphatase that removes the inhibitory phosphate from TAF_I_110. These results identify hTAF_I_110 as a novel substrate of hCdc14B, revealing that hCdc14B counteracts Cdk1/cyclin B-mediated phosphorylation of SL1.

To examine whether hCdc14B is capable to overcome mitotic repression of Pol I transcription, we performed *in vitro* transcription assays using extracts from M-phase cells. As reported before [18, 19], extracts from mitotic cells are transcriptionally inactive (S1D Fig). Transcriptional repression was relieved if extracts were depleted from Cdk1/cyclin B by bead-bound p13suc1, underscoring the importance of Cdk1/cyclin B in mitotic repression of Pol I transcription (Fig 1D, lanes 1, 2). Transcriptional activity was also restored if transcription was performed in conditions that prevent Cdk1/cyclin B-dependent phosphorylation and thus inactivation of SL1. If ATP was replaced by the non-hydrolysable ATP analogue AMP-PNP (adenylyl-imidotriphosphate) or by inclusion of the kinase inhibitor DMAP (6-dimethylaminopurine), transcriptional repression was relieved (Fig 1D, lanes 3–10 and S1E Fig). Addition of calf intestine alkaline phosphatase (CIAP) or Cdc14B relieved transcriptional repression in ATP-containing reactions, reinforcing that Cdc14B-dependent dephosphorylation of TAF_I_110 at T852 reactivates Pol I transcription at the exit from mitosis.

Nucleoli disassemble during mitosis and many nucleolar proteins are released into the cytoplasm [3]. However, UBF remains bound to rDNA, thus bookmarking rDNA for resumption of transcription upon mitotic exit (Fig 1E and S1F Fig; see also ref. [22]). Both in yeast and mammals, Cdc14B is sequestered in nucleoli during interphase and activated both during mitosis and DNA damage upon release from nucleolar chromatin [20, 23]. In accord with these observations, we found that in asynchronous cells hCdc14B was preferentially bound to intergenic spacer sequences separating individual rDNA repeats (5’- and 3’-IGS). Binding of hCdc14B to rDNA was abrogated in mitotic cells (Fig 1E, S1F and S1G Fig), reinforcing that Cdc14B is inactivated during interphase by confinement to the intergenic spacer, and is released from nucleolar chromatin during mitosis. Significantly, UBF and histone H3 remained associated with rDNA in M-phase cells. Together, the results suggest that release from rDNA enables hCdc14B to dephosphorylate SL1, a step that is required for resumption of rDNA transcription when cells re-enter the cell cycle.

### Phosphorylation of T852 is not sufficient for mitotic inactivation of SL1

**Fig 2:**
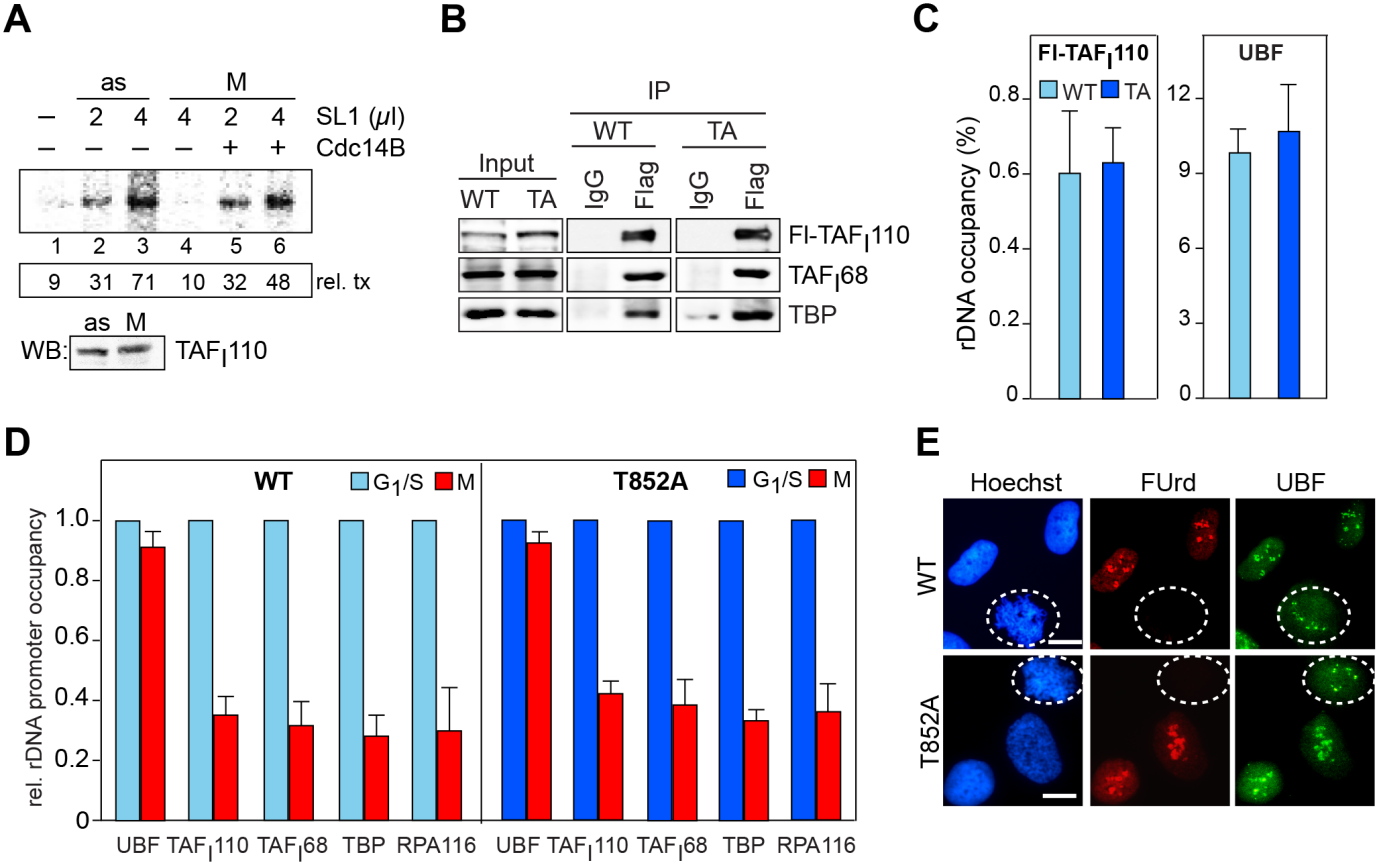
Reactivation of mitotic SL1 by Cdc14B. (A) Transcriptional activity of mitotic SL1 is increased by hCdc14B. SL1 was immunopurified from asynchronous (as) or nocodazole-arrested (M) HeLa cells and transcriptional activity was assayed in a reconstituted SL1-responsive transcription system in the presence or absence of GST-hCdc14B. The numbers below indicate the relative amount of run-off transcripts quantified by PhosphorImaging (rel. tx.). The Western blot below shows that comparable amounts of SL1 from asynchronous and mitotic cells were used in the transcription assays. (B) Mutation of T852 does not impair formation of SL1 complexes. Flag-hTAF_I_110/WT or Flag-hTAF_I_110/T852 was immunoprecipitated from Hela cells stably expressing wildtype or mutant TAF_I_110 and co-precipitated hTAF_I_68 and TBP were analyzed on immunoblots with anti-TAF_I_68 and anti-TBP antibodies. The input lanes contain 10–30 μg of nuclear lysate. (C) Mutation of T852 does not affect SL1 binding to the rDNA promoter. ChIP of Flag-tagged TAF_I_110 and UBF from HeLa cells expressing wildtype Flag-hTAF_I_110 (light blue bars) or mutant Flag-hTAF_I_110/T852 (dark blue bars). Precipitated DNA was analyzed by qPCR using primers that amplify the rDNA promoter. Bars denote the mean value (± SD) from two biological replicates. (D) Mutation of T852 does not prevent loss of SL1 from the rDNA promoter during mitosis. Left panel: ChIP of UBF, SL1 (TBP, TAF_I_110, TAF_I_68), and Pol I (RPA116) from G_1_/S phase-arrested (light blue bars) or M-phase (red bars) HeLa cells expressing Flag-tagged hTAF_I_110/WT. Right panel: ChIPs from G_1_/S- and M-phase HeLa cells expressing Flag-tagged hTAF_I_110/T852A (dark blue and red bars). Precipitated DNA was analyzed by qPCR using primers that amplify the rDNA promoter. Bars denote the mean value (± SD) from two biological replicates. (E) Nucleolar transcription is repressed in mitotic cells regardless of whether they express wildtype (WT) or mutant hTAF_I_110 (T852A). Cells were labeled for 20 min with FUrd and nucleolar transcripts were visualized with antibodies against BrdU. NORs were immunostained with anti-UBF antibodies. Mitotic cells are encircled. Representative images out of 200 analyzed cells are shown. Scale bar, 10 μm. For quantification of the fluorescence signals, see S2B Fig.

To prove that hCdc14B-dependent dephosphorylation of SL1 is required to activate rDNA transcription at the exit from mitosis, we assayed the activity of immunopurified SL1 in a reconstituted transcription system. SL1 from asynchronous cells stimulated transcription up to 8-fold (Fig 2A, lane 3), while the same amount of SL1 from mitotic cells was inactive (Fig 2A, lane 4). The activity of mitotic SL1 was restored by addition of hCdc14B, demonstrating that dephosphorylation of T852 by hCdc14B relieves Cdk1/cyclin-dependent mitotic inactivation of SL1 (Fig 2A, lanes 5, 6).

If Cdk1/cyclin B-mediated phosphorylation of TAF_I_110 is the only mechanism that inactivates SL1 and represses Pol I transcription during mitosis, then mutation of T852 should prevent mitotic inactivation of SL1. To test this, we established HeLa cell lines that stably overexpress Flag-tagged wildtype TAF_I_110 or a mutant in which T852 has been replaced by alanine (TAF_I_110/T852A). Both wildtype and mutant TAF_I_110 assembled into proper TBP-TAF complexes, indicating that replacement of T852 by alanine does not affect the interaction of TAF_I_110 with other SL1 subunits (Fig 2B). Moreover, both wildtype and mutant TAF_I_110 bound with similar efficiency to the rDNA promoter, supporting that phosphorylation of T852 has no impact on DNA binding (Fig 2C). To further corroborate these results, we compared rDNA occupancy of Pol I, UBF and SL1 in synchronized HeLa cells expressing Flag-TAF_I_110 or Flag-TAF_I_110/T852A. While UBF remained associated with rDNA throughout the cell cycle [22, 24], binding of SL1 and Pol I was decreased in mitotic cells (Fig 2D). Decreased rDNA occupancy of Pol I and SL1 was observed in mitotic cells regardless of whether wildtype or mutant TAF_I_110 were overexpressed, indicating that the assembly of Pol I pre-initiation complexes was impaired both in cells overexpressing wildtype TAF_I_110 or mutant TAF_I_110/T852A.

To confirm that rDNA transcription is switched-off during mitosis in cells expressing mutant TAF_I_110, we pulse-labeled nascent RNA with FUrd and visualized nascent transcripts that co-localize with UBF at mitotic NORs (Fig 2E). Similar amounts of FU-labeled transcripts were synthesized in interphase cells, regardless of whether wildtype or mutant TAF_I_110 was expressed. Surprisingly, though the level of ectopic TAF_I_110/T852A was about 2–3 fold higher than that of endogenous TAF_I_110 (S2A Fig), no nascent transcripts were visible in mitotic cells expressing mutant TAF_I_110/T852A. The observation that the phosphorylation-deficient mutant repressed rDNA transcription as efficiently as wildtype TAF_I_110 reveals that phosphorylation of TAF_I_110 by Cdk1/cyclin B is necessary but not sufficient for mitotic repression of rDNA transcription.

### TAF_I_68 is hypoacetylated during mitosis

**Fig 3:**
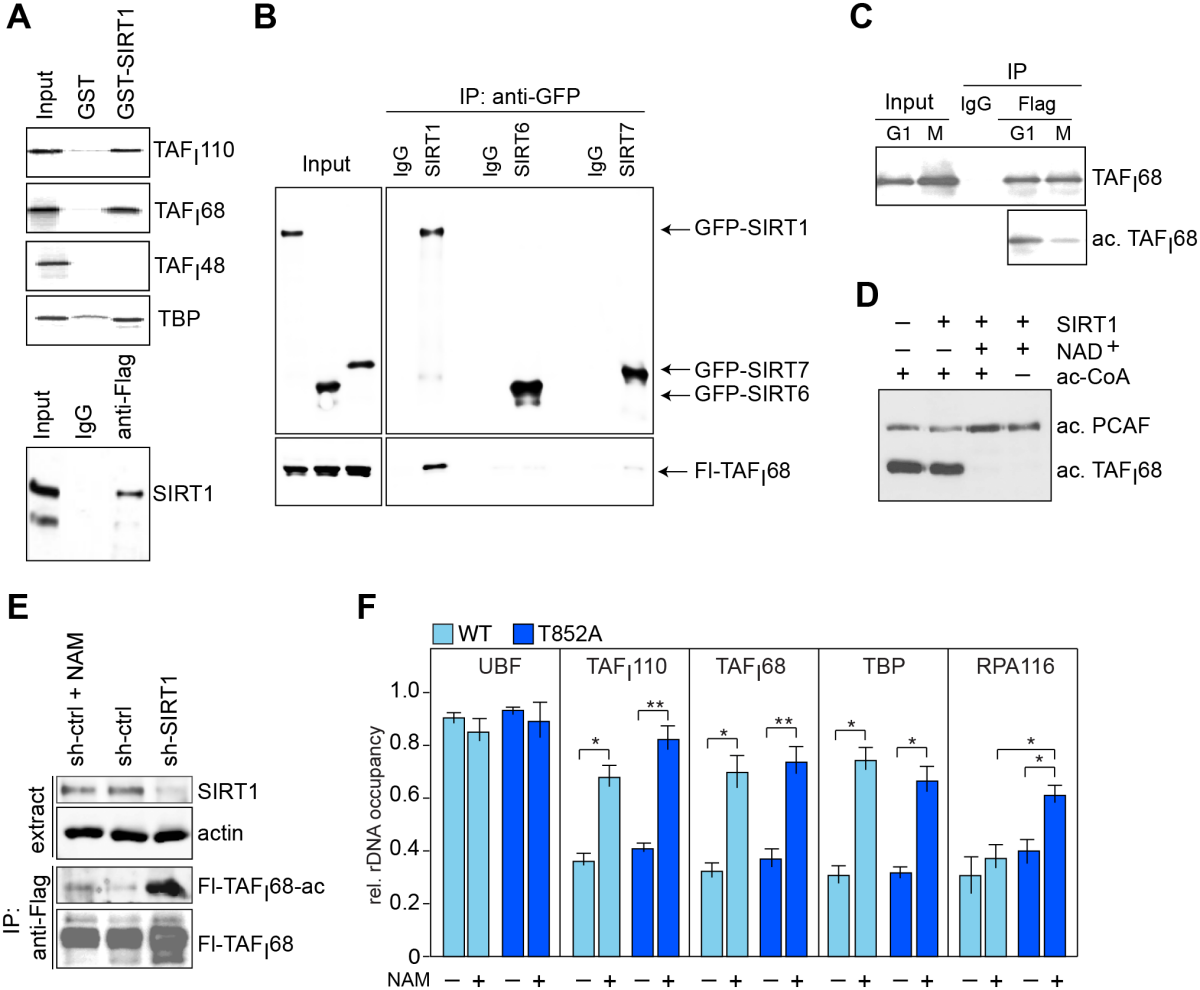
SIRT1 deacetylates TAF_I_68 in early mitosis. (A) SIRT1 interacts with SL1. Upper panels: Pull-down experiment using immobilized GST or GST-SIRT1 and the indicated ^35^S-labeled proteins. Bound proteins were analyzed by gel electrophoresis. The input lanes show 10% of proteins used for pull-down. Lower panel: Western blot showing co-immunoprecipitation of SIRT1 with Flag-hTAF_I_68. 2.5% of nuclear extract proteins (Input) and 95% of precipitated proteins were loaded. (B) TAF_I_68 interacts with SIRT1, but not with SIRT6 and SIRT7. Flag-hTAF_I_68 was co-expressed in HEK293T cells with GFP-tagged SIRT1, SIRT6, or SIRT7. GFP-tagged proteins were bound to GFP-Trap, and co-precipitated Flag-hTAF_I_68 was monitored on Western blots using anti-TAF_I_68 antibodies (lower panel); SIRTs were monitored with anti-GFP antibodies (upper panel). (C) TAF_I_68 is hypoacetylated during mitosis. HeLa-Flag-hTAF_I_110 cells were synchronized at G_1_/S (G1) by thymidine or in prometaphase (M) by nocodazole treatment. TAF_I_68 was immunoprecipitated and analyzed on Western blots using anti-TAF_I_68 (10% of IP loaded) or anti-Ac-K-specific antibodies (90% of IP loaded). (D) SIRT1 deacetylates TAF_I_68 *in vitro*. Flag-TAF_I_68 expressed and pre-acetylated in Sf9 cells by co-expression with PCAF was incubated with GST-SIRT1 in the absence or presence of 0.5 μM NAD^+^. Acetylation of TAF_I_68 was monitored on Western blots using anti-acetyl-lysine antibodies. (See also ref. [21]). (E) SIRT1 deacetylates TAF_I_68 at early mitosis. Western blot showing acetylation of Flag-TAF_I_68 in nocodazole-treated cells cultured in the absence or presence of nicotinamide (+/-NAM) and in cells depleted of SIRT1 (shSIRT1). The input level of SIRT1 and immunopurified Flag-hTAF_I_68 is shown. (F) Inhibition of SIRT1 prevents mitotic release of SL1 from rDNA. ChIP showing rDNA promoter occupancy of UBF, SL1 (TAF_I_68, TAF_I_110, TBP), and Pol I (RPA116) in nocodazole-treated HeLa cells expressing Flag-tagged wildtype (WT, light blue bars) or mutant (T852A) hTAF_I_110 (dark blue bars). The graphs depict the relative occupancy in untreated or NAM-treated cells (5 mM, 5 h). The mean values (±SD) from four biological replicates are shown (*p≤ 0.05; **p≤ 0.01).

As phosphorylation of TAF_I_110 was not sufficient for mitotic inactivation of SL1, we reasoned that other posttranslational modifications contribute to inactivation of SL1 at the entry into mitosis. Previous work has established that TAF_I_68 (TAF1B), a TBP-associated factor that is structurally related to the general transcription factor TFIIB [25], is acetylated by the histone acetyltransferase PCAF at two lysine residues, K438 and K443 [26]. Acetylation was shown to augment the DNA-binding activity of TAF_I_68 and activate rDNA transcription [21]. Mutation of both lysine residues abolished PCAF-dependent acetylation of TAF_I_68 (S3A and S3B Fig). The correlation between acetylation and DNA binding efficiency of TAF_I_68 implies that deacetylation of TAF_I_68 impairs transcription complex assembly. In a previous study we have shown that PCAF-dependent acetylation of TAF_I_68 was counteracted by SIRT1 [21], the founding member of the Sirtuin family of NAD^+^-dependent histone deacetylases. In support of SIRT1 interacting with SL1, immobilized SIRT1 associated with TAF_I_110, TAF_I_68 and TBP, subunits of the SL1 complex (Fig 3A, top). Consistently, endogenous SIRT1 was co-immunoprecipitated with ectopic TAF_I_68 (Fig 3A, bottom). No association of TAF_I_68 was observed with either SIRT6 or SIRT7, highlighting the specificity of TAF_I_68 binding to SIRT1 (Fig 3B). This result reveals that the two nucle(ol)ar Sirtuins SIRT1 and SIRT7 serve opposing functions, SIRT7 activating rDNA transcription in cycling cells [27, 28], while deacetylation of TAF_I_68 by SIRT1 being required for mitotic repression of rDNA transcription. Accordingly, acetylation of TAF_I_68 was decreased in prometaphase- compared to G_1_/S-arrested cells, supporting that cell cycle-dependent fluctuations of SL1 acetylation are involved in mitotic repression of rRNA synthesis (Fig 3C). Consistent with SIRT1 targeting TAF_I_68, *in vitro* deacetylation of TAF_I_68 by SIRT1 required the presence of NAD^+^ (Fig 3D). Likewise, *in vivo* acetylation of TAF_I_68 was increased if cells were treated with nicotinamide (NAM), a competitive inhibitor of NAD^+^-dependent deacetylases (Fig 3E). Knockdown of SIRT1 further increased acetylation, proving that SIRT1 rather than another member of the Sirtuin family deacetylates TAF_I_68.

Next, we examined whether SIRT1-dependent deacetylation of TAF_I_68 contributes to mitotic inactivation of SL1. For this, we compared rDNA occupancy of SL1 (TBP, TAF_I_110 and TAF_I_68), Pol I (RPA116) and UBF in prometaphase cells expressing wildtype or mutant TAF_I_110 (Fig 3F). As expected, rDNA occupancy of UBF was comparable in both cell lines and was not affected by NAM-dependent inhibition of SIRT1 activity. In contrast, treatment with NAM similarly increased binding of SL1 regardless of whether cells expressed wildtype or mutant TAF_I_110. This indicates that deacetylation of TAF_I_68 by SIRT1 rather than phosphorylation of TAF_I_110 weakens the association of SL1 with the rDNA promoter, which leads to partial displacement of SL1 from rDNA in early mitosis. Consistent with acetylation of TAF_I_68 being required for binding of SL1 and transcription complex formation, rDNA promoter occupancy of Pol I was elevated in NAM-treated mitotic TAF_I_110/T852A cells, indicating that under these conditions transcriptional repression was partially relieved. Thus, unphosphorylated TAF_I_110 and acetylated TAF_I_68 are required for the assembly of productive Pol I transcription initiation complexes. Together, these results imply that mitotic repression of Pol I transcription is brought about by a dual mechanism. Deacetylation of TAF_I_68 by SIRT1 weakens the association of SL1 with rDNA, while phosphorylation of TAF_I_110 by Cdk1/cyclin B impairs the interaction with UBF, leading to mitotic repression of, rDNA transcription.

### Inhibition of SIRT1 bypasses mitotic repression of Pol I transcription

**Fig 4:**
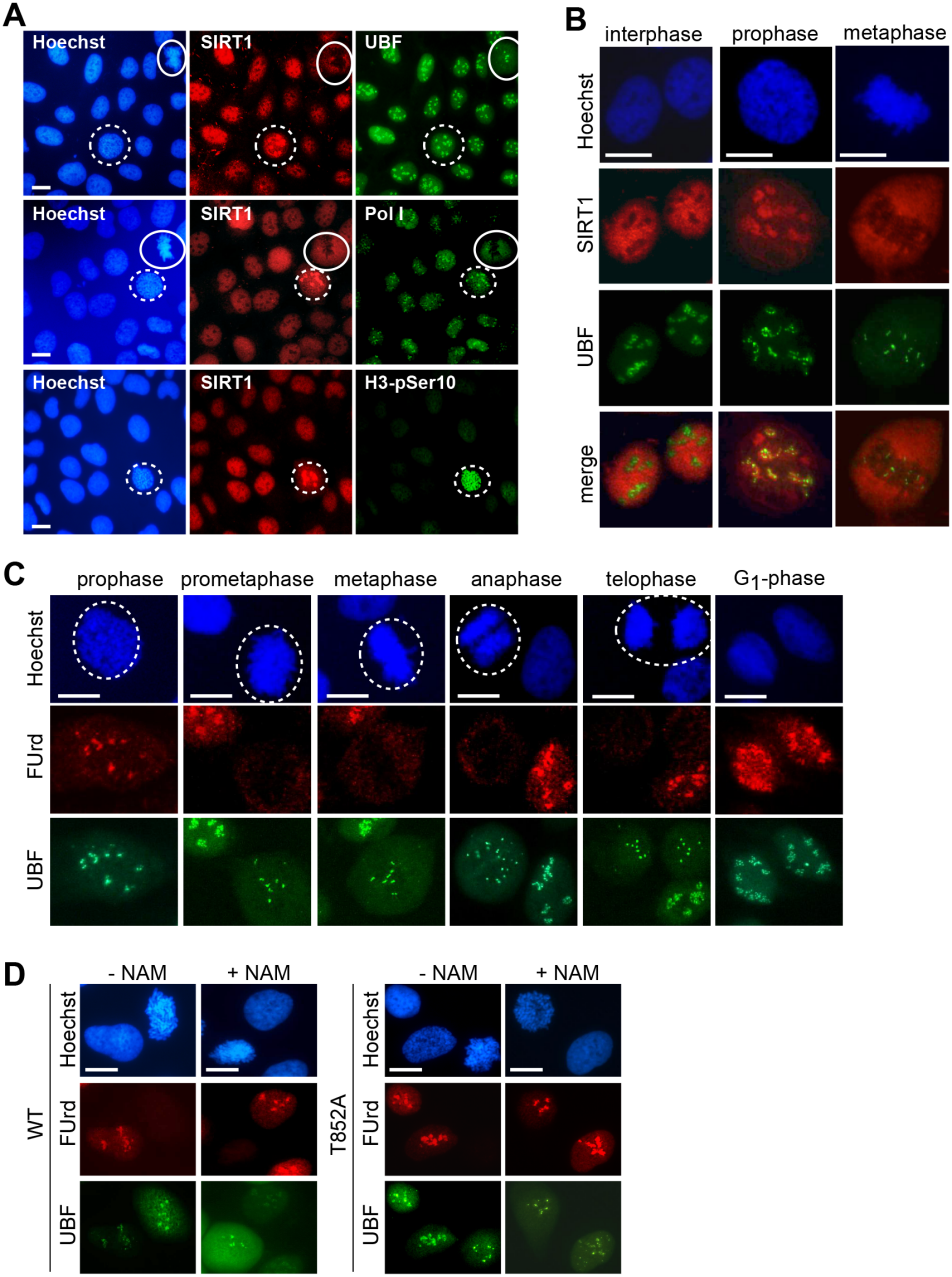
Both phosphorylation of TAF_I_110 and deacetylation of TAF_I_68 are necessary for mitotic inactivation of SL1. (A) SIRT1 translocates to nucleoli during prophase. Immunostaining of SIRT1, UBF, Pol I, and histone H3 phosphorylated at serine 10 (H3-pSer10) in U2OS cells is shown. Prophase cells are encircled by a dotted line, metaphase cells by a solid line. Representative images out of 100 analyzed mitotic cells are shown. Scale bars, 7 μm. See also S4 Fig. (B) SIRT1 co-localizes with UBF at the onset of mitosis. Confocal immunofluorescence microscopy of selected cells from (A) showing co-localization of SIRT1 (red) and UBF (green) in prophase, but not in metaphase and interphase cells. Representative images out of 24 mitotic cells are shown. Scale bars, 10 μm. See also S4 Fig. (C) Pol I transcription is repressed from prometaphase to telophase. FUrd-labeled nascent RNA was visualized by immunofluorescence (FUrd, red), NORs by immunostaining of UBF (green) and chromatin by staining with Hoechst 33342. Mitotic cells are encircled. Representative images out of 200 analyzed cells are shown. Scale bars, 10 μm. See also S4 Fig. (D) Inhibition of SIRT1 prevents mitotic repression of Pol I transcription in cells expressing TAF_I_110/T852A. HeLa cells expressing hTAF_I_110/WT or hTAF_I_110/T852A were left untreated or treated with NAM (5 mM, 5 h) before pulse-labeling with FUrd. Nascent transcripts were visualized by immunostaining with anti-BrdU antibody (red); NORs were visualized with anti-UBF antibodies (green). Approximately 100 NORscells were analyzed from each cell line; representative images are shown. Scale bars, 10 μm. See also S4 Fig.

In asynchronous cells the majority of SIRT1 resides in the nucleoplasm and is excluded from nucleoli. In prophase cells, however, SIRT1 transiently localizes in nucleoli, co-staining with UBF and Pol I (Fig 4A, dashed circles). Prophase cells have an intact nuclear envelope but show chromosome condensation and are positive for the mitotic histone mark H3-pSer10. Consistent with UBF bookmarking mitotic NORs [22, 29], UBF remained bound to NORs throughout mitosis. Co-localization of SIRT1 and UBF was confined to prophase and was not detected at later stages of mitosis (Fig 4B). Enrichment of SIRT1 at NORs preceded repression of Pol I transcription, monitored by visualization of NOR-associated nascent RNAs at different stages of mitosis. Consistent with Pol I transcription being switched-off during mitosis, no nascent RNA was detected at UBF-specific foci from prometaphase to telophase (Fig 4C).

Next we monitored nascent pre-rRNA levels in mitotic HeLa cells expressing Flag- TAF_I_110/WT or TAF_I_110/T852A in the absence or presence of the Sirtuin inhibitor NAM. The rationale of this experiment was to find out whether mitotic inactivation of SL1 would be relieved and pre-rRNA synthesis restored if both phosphorylation of T852 and deacetylation of TAF_I_68 were prevented. Immunofluorescence analysis of fluorouridine (FUrd)-labeled RNA revealed low but significant levels of nascent transcripts at mitotic NORs in NAM-treated cells that express TAF_I_110/T852A (Fig 4D). In contrast, there was no nascent RNA visible at mitotic NORs of HeLa cells expressing wildtype hTAF_I_110, regardless of whether the cells were treated with NAM or not. The finding that SL1 was not inactivated if both Cdk1/cyclin B-dependent phosphorylation of hTAF_I_110 and SIRT1-dependent deacetylation of TAF_I_68 were prevented highlight the functional significance of both activities in mitotic repression of Pol I transcription.

## Discussion

At mitosis, there is substantial reorganization of chromosomal architecture as cells prepare to exit the G2-phase of the cell cycle and enter the prophase of mitosis. This reorganization of nuclear structure is accompanied by a global shut-off of transcriptional activity. Transcription by all three classes of nuclear DNA-dependent RNA polymerases stops by mid-prophase and resumes in late telophase [1, 2]. Mitotic repression of rDNA transcription correlates with perturbation of nucleolar structure and dispersion of most nucleolar proteins. However, basal factors required for transcription initiation are maintained on metaphase chromosomes [3, 12, 24, 30], thus marking rRNA genes for rapid assembly of pre-initiation complexes and resumption of rRNA synthesis in G_1_-phase. Mitotic repression of Pol I transcription is brought about by phosphorylation of TAF_I_110, the large subunit of the basal Pol I-specific transcription factor SL1 by Cdk1/cyclin B [13, 18]. Phosphorylation of TAF_I_110 at threonine 852 impairs the capability of SL1 to interact with UBF, thereby abrogating the assembly of transcription-competent initiation complexes [13]. Here we show that phosphorylation of TAF_I_110 at threonine 852 is counteracted by the phosphatase Cdc14B, which regulates progression through mitosis [20]. Cdc14B is sequestered during interphase in the nucleolus by association with intergenic spacer sequences that separate individual rDNA transcription units. At prometaphase, Cdc14B is released from rDNA, allowing dephosphorylation of TAF_I_110 and resumption of rRNA synthesis in early G_1_-phase [19]. These results suggested that Cdc14B-dependent dephosphorylation of TAF_I_110 is the molecular switch that reactivates SL1 at the exit from mitosis. Surprisingly, however, transcription was also repressed in mitotic cells that express the phosphorylation-deficient mutant TAF_I_110/T852A, indicating that phosphorylation of T852 by Cdk1/cyclin B is not the only mechanism that inactivates SL1 during mitosis.

**Fig 5:**
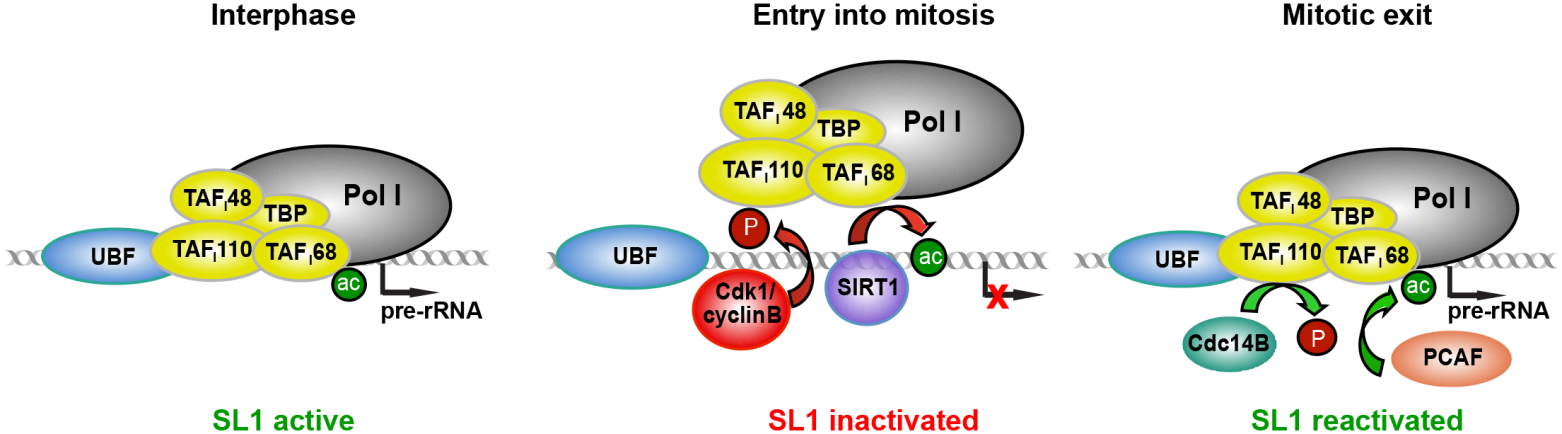
Model illustrating inactivation of SL1 and mitotic repression of Pol I transcription. In interphase cells, TAF_I_68 is acetylated by PCAF, which promotes binding of SL1 to the rDNA promoter. At the entry into mitosis, SIRT1 deacetylates TAF_I_68, deacetylation weakening the interaction of SL1 with rDNA. Mitotic repression is reinforced by Cdk1/cyclin B-dependent phosphorylation of TAF_I_110 at T852, which impairs the interaction between SL1 and UBF. At the end of mitosis, SL1 activity is restored by Cdc14B-mediated dephosphorylation of T852 and *de novo* acetylation of TAF_I_68.

In addition to phosphorylation of TAF_I_110, repression of rDNA transcription upon entry into mitosis involves deacetylation of another SL1 subunit, TAF_I_68, by the NAD^+^-dependent deacetylase SIRT1. TAF_I_68 is acetylated by the histone acetyltransferase PCAF, acetylation promoting the association of SL1 with the rDNA promoter [21]. The functional significance of TAF_I_68 acetylation, however, remained obscure. We found that mitotic repression of transcription was alleviated in the presence of nicotinamide, a competitive inhibitor of NAD^+^-dependent deacetylases. Moreover, PCAF-dependent acetylation of TAF_I_68 was counteracted by SIRT1, which is transiently enriched in nucleoli at prophase. This identifies TAF_I_68 as novel substrate of SIRT1, SIRT1-dependent deacetylation of SL1 reinforcing mitotic shut-off of Pol I transcription. In addition, SIRT1 is known to deacetylate the euchromatic histone mark H4K16Ac, and to facilitate loading of histone H1 and the condensin I complex, which promotes facultative heterochromatin formation and thereby contributes to chromosome integrity and stability during mitosis [31–33]. Our finding that SIRT1 deacetylates TAF_I_68 is in accord with numerous studies demonstrating that the deacetylase activity of SIRT1 targets histones, chromatin regulators, and a world of nonhistone substrates, including metabolic enzymes, transcription factors, cytoskeleton proteins, and many others [34]. Like other transcription factors, such as p53, acetylation of TAF_I_68 increases site-specific DNA binding activity. Accordingly, deacetylation by SIRT1 weakens the association of SL1 with rDNA [21]. Nucleolar enrichment of SIRT1 and decreased rDNA transcription correlates with increased dynamics of the Pol I transcription machinery at mitotic NORs determined by FRAP measurements [24, 35]. Thus, cells regulate rDNA transcription complex formation by reversible acetylation of TAF_I_68, acetylation of TAF_I_68 increasing and deacetylation by SIRT1 decreasing DNA binding of SL1. At the entry into mitosis two posttranslational modifications, i.e., phosphorylation of TAF_I_110 and deacetylation of TAF_I_68, inactivate SL1, thereby attenuating pre-initiation complex formation (Fig 5). These results reveal that fine-tuned reversible acetylation and phosphorylation of TAF_I_s is an effective means to regulate SL1 activity and mediate fluctuations of Pol I transcription during cell cycle progression.

## Materials and Methods

### Cell culture, transfections, and cell treatments

Cells cultured according to standard conditions (ATCC) were transfected with Fugene6, Lipofectamin 2000 (Invitrogen), or calcium phosphate. Clonal HeLa cell lines that stably express Flag-tagged wildtype hTAF_I_110 or mutant hTAF_I_110/T852A were selected in the presence of G418 (750 μg/ml). To knockdown SIRT1, cells transfected with specific shRNA expression plasmids (Sigma) were selected in the presence of puromycin (1 μg/ml) and analyzed after 5–6 days. HeLa and U2OS cells were synchronized at G_1_/S with thymidine (2 mM, 23 h), released for 8 h, and arrested in prometaphase with nocodazole (80 ng/ml). To inhibit SIRT1 activity, cells were treated for 5 h with 5–10 mM nicotinamide (NAM).

### Plasmids and antibodies

Plasmids encoding SIRT1, SIRT6, SIRT7, hCdc14B, TBP, and individual TAF_I_s have been described [15, 20, 21, 27, 28]. cDNA encoding hTAF_I_110 (accession number NM_005679) was cloned into pRc/CMV (Invitrogen). Threonine at position 852 of hTAF_I_110, and lysine residues at position 438 and 443 of hTAF_I_68 were converted into alanine or arginine by site-directed mutagenesis. Primers used for PCR-mediated mutagenesis are listed in S2 Table. Antibodies against UBF [36], RPA194 and RPA116 [37, 38] and TAF_I_s [15] have been described. Anti-TBP antibodies (clone 3G3) were provided by Laszlo Tora. Antibodies against the Flag epitope (F3165), actin, BrdU (BU-33) were from Sigma, anti-acetylated lysine from Cell Signaling (9441), anti-UBF (sc-13125) and anti PCAF (H-369) from Santa Cruz, anti-SIRT1 (07–131), anti-histone H3-pSer10 (06–570) from Millipore, anti-GFP from Abcam, anti-histone H3 from Diagenode and anti-Cdc14B from Zymed.

### Immunocytochemistry and nuclear run-on assays

Immunofluorescence was performed as described [39]. The secondary antibodies were conjugated to Cy2, Cy3, or FITC (Dianova), Alexa Fluor 488 or Alexa Fluor 555 (Molecular Probes). To visualize nascent RNAs, cells grown on poly-L-lysine coated coverslips were labeled with 2 mM fluorouridine (FUrd) for 20 min, fixed with 2% paraformaldehyde, permeabilized with methanol, incubated with the respective antibodies and stained with fluorophore-coupled secondary antibodies. DNA was stained with Hoechst 33342.

### Microscopy, image acquisition and quantitative analysis

Images were visualized with a Zeiss microscope (Axiophot) using a 40×1.3 oil immersion Plan-Neofluar magnifying objective (Carl Zeiss), captured with a device camera (DS-Qi1Mc; Nikon) and processed with NIS-Elements software (version BR 3.10; Nikon). Images were quantified images using ImageJ and calculated as described [40]. Confocal laser scanning microscopy (CLSM) was done with LSM META 510 (Zeiss). Immunofluorescence images were quantified using ImageJ as described [40].

### Expression and purification of recombinant proteins

HEK293T or HeLa cells expressing epitope-tagged proteins were lysed in buffer AM-600 (600 mM KCl, 20 mM Tris-HCl [pH 7.9], 5 mM MgCl_2_, 0.2 mM EDTA, 10% glycerol, 0.5 mM DTT) supplemented with 0.1% NP-40, protease inhibitors (Roche Complete, PMSF), and HDAC inhibitors (500 nM TSA, 5 mM sodium butyrate, 10 mM nicotinamide). Lysates were incubated for 4 h at 4°C with mouse or rabbit IgGs bound to protein A/G-agarose (Roche, Dianova) or with M2 anti-Flag beads (Sigma). After washing in lysis buffer, tagged proteins were eluted in buffer AM-300 supplemented with 0.1% NP-40 and Flag-peptide (20 μg/100 μl). GST-tagged proteins were expressed in *E*. *coli* and affinity-purified on Glutathione-Sepharose 4B (GE healthcare). Flag-tagged UBF was isolated from Sf9 cells [36]. GFP-tagged proteins were bound to GFP-Trap (ChromoTek) in buffer AM-300 containing 0.1% NP-40, Roche complete and HDAC inhibitors. Flag-tagged UBF and PCAF were isolated from baculovirus-infected Sf9 cells as described [36, 21].

### Protein pull-down experiments and co-immunoprecipitation

2 μg of GST or GST-tagged proteins immobilized on Glutathione-Sepharose 4B were incubated with *in vitro* synthesized ^35^S-labeled proteins for 4 h at 4°C in 120 mM KCl, 20 mM Tris-HCl [pH 7.9], 5 mM MgCl_2_, 0.2 mM EDTA, 10% glycerol, 0.2% NP-40 and protease inhibitors (Roche Complete, PMSF). After washing, bead-bound proteins were analyzed by SDS-PAGE and PhosphorImaging. Endogenous SL1 was immunopurified from HeLa nuclear extracts with anti-TBP antibody (clone 3G3) to Dynabeads, mouse IgGs were used as control [15]. To monitor the interaction of Flag-tagged hTAF_I_68 with GFP-tagged Sirtuins, cells were lysed in buffer containing 300 mM KCl, 20 mM Tris-HCl [pH 7.9], 5 mM MgCl_2_, 0.2 mM EDTA, 0.5% Triton X-100 and protease inhibitors (Roche Complete, 0.5 mM PMSF). After capture on GFP-trap beads (4 h, 4°C), associated hTAF_I_68 was monitored on Western blots. To examine the association of TAF_I_110 with Cdc14B, Flag-tagged hTAF_I_110 co-expressed with GFP-hCdc14B in HEK293T cells was bound to M2-agarose in the same buffer containing 120 mM KCl, and co-purified GFP-hCdc14B was visualized on immunoblots. Co-immunoprecipitation of Flag-tagged hTAF_I_110 with endogenous TAF_I_68 and TBP was performed in the presence of 250 mM KCl.

### In vitro transcription assays

In vitro transcription reactions contained 40 ng of linearized plasmid pHrP_2_ comprising human rDNA sequences from -410 to +378 (with respect to the transcription start site) and 40 μg of extract from HeLa cells [13]. Depletion of Cdk1/cyclin B from mitotic extracts with immobilized p13suc1 has been described [18]. SL1 was immunopurified from extracts with anti-TBP (3G3) antibodies immobilized on anti-mouse IgG Dynabeads. Flag-tagged UBF was immunopurified from baculovirus-infected Sf9 cells [37]. The SL1-responsive reconstituted transcription system contained 5 μl of Pol I (MonoS fraction), 3 μl of TIF-IA (Q-Sepharose fraction) [41], and 10 ng of UBF. After incubation for 1 h at 30°C, transcripts were purified and analyzed by gel electrophoresis and PhosphorImaging.

### Chromatin immunoprecipitation (ChIP) assays

Cells were fixed with 1% formaldehyde (10 min, RT), quenched with 0.125 M glycine, and sonicated to yield 250–500 bp DNA fragments. After 5-fold dilution with IP dilution buffer (0.01% SDS, 1.1% Triton X-100, 1.2 mM EDTA, 16.7 mM Tris-HCl [pH 8.0], 167 mM NaCl) and preclearing with protein A/G Sepharose, lysates were incubated overnight with the respective antibodies, and protein-DNA complexes were captured on protein A/G Sepharose followed by washes in low salt buffer (150 mM NaCl, 50 mM Tris-HCl [pH 8.0], 5 mM MgCl_2_, 1% Triton X-100), high salt buffer containing 500 mM NaCl, followed by LiCl buffer (250 mM LiCl, 10 mM Tris-HCl [pH 8.0], 5 mM EDTA, 0.5% Na-deoxycholate, 0.5% Triton X-100) and TE buffer. After reversal of the crosslink and proteinase K digestion, DNA was purified and analyzed by qPCR (Roche LightCycler480). Precipitated DNA was calculated as the percentage of DNA in the immunoprecipitates compared to input DNA. The primers used for PCR are listed in S1 Table.

### Phosphopeptide mapping of TAF_I_110

Recombinant TAF_I_110 was immunopurified from HEK293T cells overexpressing FLAG-tagged TAF_I_110 and radiolabeled *in vitro* by incubation with extracts from mitotic HeLa cells or immobilized Cdk1/cyclin B in the presence of ^32^P-ATP. After digestion overnight at 37°C with trypsin (5 μg, Promega, sequencing grade) in 50 mM ammonium bicarbonate and lyophilisation, the peptides were resolved on cellulose thin-layer plates by electrophoresis for 25 min at 1000V in 1% (w/v) ammonium carbonate (pH 8.9), followed by ascending chromatography in a buffer containing 62.5% isobutyric acid, 1.9% n-butanol, 4.8% pyridine, and 2.9% acetic acid [13, 41]. Phosphoamino acid analysis was performed according to Boyle et al. [42].

## Supporting Information

S1 Fig Mitotic phosphorylation of hTAF_I_110 at T852 represses Pol I transcription. (A) Flag-hTAF_I_110 was phosphorylated *in vitro* with ^32^P and immunopurified Cdk1/cyclin B in the absence or presence of the Cdk1/cyclin B inhibitor roscovitine, and subjected to acid hydrolysis and phosphoamino acid analysis. The letters at the right indicate the position of phosphoserine (S), phosphothreonine (T), and phosphotyrosine (Y). (B) Sequence of the synthetic tryptic phosphopeptide (marked in yellow) encompassing the carboxy-terminal region of human TAF_I_110 (UniProtKB accession number Q15572). The peptide was used for co-migration with ^32^P-labeled tryptic phosphopeptides of hTAF_I_110 in 2-dimensional tryptic peptide maps (see Fig 1A). (C) Threonine 852 is conserved among mammals. Amino acid sequence alignment of the carboxyterminal region of TAF_I_110/TAF_I_95/TAF1C from human, mouse, rat, horse, dog, and hedgehog, showing conservation of threonine 852. The UniProtKB or NCBI accession numbers are indicated in brackets. (D) Mitotic repression of rDNA transcription is relieved by the ATP analogue AMP-PNP. Transcription by Pol I was assayed *in vitro* using 40 μg of extracts from asynchronous (as) or nocodazole-arrested mitotic (M) HeLa cells and 40 ng of the reporter template pHrP_2_ linearized with Nde I. Reactions contained either 200 μM ATP or 200 μM AMP-PNP. (E) Cdc14B counteracts Cdk1-mediated mitotic repression of Pol I transcription. Transcriptional activity was assayed in extracts from mitotic HeLa cells in the presence of ATP (200 μM)-/+ 2.5 mM DMAP, or the non-hydrolysable analog AMP-PNP. Where indicated, the assays were supplemented with similar units of calf intestine phosphatase (CIAP) or purified GST-hCdc14B. Run-off transcripts were analyzed on native polyacrylamide gels and visualized by PhosphorImaging. An internal control demonstrates equal loading. (F) Ccd14B is released from rDNA during mitosis. ChIP of Cdc14B and UBF from asynchronous (as) or nocodazole-treated (M) HeLa cells. Increasing amounts of precipitated DNA were analyzed by semi-quantitative PCR, using the indicated primer pairs (listed in S1 Table) and labeling the PCR products with ^32^P-dCTP. A scheme presenting part the human rDNA repeat unit and the position of the PCR primers is shown above. The arrow indicates the transcription start site, black boxes the regions encoding 18S, 5.8S and 28S rRNA, the thin line the intergenic spacer (IGS). (G) Evaluation of the specificity of the anti-Cdc14B antibody used for ChIP. The ChIP results shown in Fig 1E show the enrichment of DNA precipitated with anti-Cdc14B over rabbit IgGs at different regions of rDNA. Bars denote means ±SD from three independent biological replicates. Related to Fig 1E.
(EPS)

S2 Fig Expression levels of Flag-tagged hTAF_I_110/WT and hTAF_I_110/T852A. (A) Nuclear extracts were prepared from HeLa cell lines which stably express Flag-hTAF_I_110/WT (clone WT17) or Flag-hTAF_I_110/T852A (clone TA4). Flag-hTAF_I_110 was detected on immunoblots using anti-Flag or anti-TAF_I_110 antibodies. (B) Quantitative measurement of fluorescence signals in single interphase and mitotic cells presented in Fig 2E. The bars denote CTCF values of Hoechst, FUrd, and UBF stains.
(EPS)

S3 Fig hTAF_I_68 is acetylated at K438 and K443. (A) Sequence of the hTAF_I_68 peptide containing the acetylated lysine residues 438 and 443. (B) Mutation of K438 and K443 abolishes acetylation by PCAF. Flag-tagged wildtype hTAF_I_68 and the point mutants K438R and K438/443R were immunopurified from HEK293T cells and acetylated with purified Flag-PCAF expressed in Sf9 cells. Acetylation of hTAF_I_68 was detected on Western blots with antibodies specific for acetylated lysine (acet. TAF_I_68). Equal amounts of TAF_I_68 in the assays and similar expression of PCAF were verified by re-probing with anti-TAF_I_68 and anti-PCAF antibodies, respectively.
(EPS)

S4 Fig Both phosphorylation of TAF_I_110 and deacetylation of TAF_I_68 are necessary for mitotic inactivation of SL1. Quantitative analyses of the fluorescence microscopy images presented in Fig 2E and Fig 4A–4D. The calculation of the corrected total cell fluorescence (CTCT) was done according to the following formula: CTCF = Integrated Density—(Area of selected cell x Mean fluorescence of background reading). Bars denote CTCF values of Hoechst, FUrd, UBF, and Pol I stains as indicated. (A) Quantitative measurement of fluorescence signals in the cells encircled in the upper and middle panel of Fig 4A. (B) Quantitative measurement of fluorescence signals in the cells presented in the three upper panels of Fig 4B. (C) Quantitative measurement of fluorescence signals in single mitotic cells and two early G_1_-phase cells from Fig 4C. (D) Quantification of fluorescence signals shown in Fig 4D.
(EPS)

S1 Table Sequences of PCR primers used in this study. The sequences of DNA oligonucleotides are shown in 5’ to 3’ orientation.
(DOCX)

S2 Table Sequences of oligonucleotides used for PCR-mediated site-directed mutagenesis. The sequences are shown in 5’ to 3’ orientation, mutated nucleotides are underlined.
(DOCX)
