# Supplementary material for: Cooperative Action of Cdk1/cyclin B and SIRT1 Is Required for Mitotic Repression of rRNA Synthesis
Source: PLoS Genet. 2015 May 29;11(5):e1005246. doi: 10.1371/journal.pgen.1005246 (PMC4449194; doi:10.1371/journal.pgen.1005246)
Supplement: S1 Table — The sequences of DNA oligonucleotides are shown in 5’ to 3’ orientation. (DOCX) [file pgen.1005246.s005.docx]

**S1 Table. Sequences of Primers used for ChIP-PCR in this study.** The sequences of DNA oligonucleotides are shown in 5’ to 3’ orientation. All primers are specific to human sequences.

| **Name** | **Primer sequence** | **Ref.** |
| --- | --- | --- |
| rDNA H42 | F: GCACCGTTTGTGTGGGGTTGG  R: CGAGACAGATCCGGCTGGCAG |  |
| rDNA H0 | F: GGAGGTATATCTTTCGCTCCGAG  R: GACGACAGGTCGCCAGAGGA |  |
| rDNA H13 | F: ACCTGGCGCTAAACCATTCGT  R: GGACAAACCCTTGTGTCGAGG | ref. [1] |
| rDNA H18 | F: GTTGACGTACAGGGTGGACTG  R: GGAAGTTGTCTTCACGCCTGA | ref. [1] |
| rDNA H27 | F: CCTTCCACGAGAGTGAGAAGC  R: TCGACCTCCCGAAATCGTACA | ref. [1] |

1. O’Sullivan AC, Sullivan GJ, McStay B. UBF biding in vivo is not restricted to regulatory

sequences within the vertebrate ribosomal repeat. Mol Cell Biol. 2002;22: 657-668.
