# Supplementary material for: Cooperative Action of Cdk1/cyclin B and SIRT1 Is Required for Mitotic Repression of rRNA Synthesis
Source: PLoS Genet. 2015 May 29;11(5):e1005246. doi: 10.1371/journal.pgen.1005246 (PMC4449194; doi:10.1371/journal.pgen.1005246)
Supplement: S2 Table — The sequences are shown in 5’ to 3’ orientation, mutated nucleotides are underlined. (DOCX) [file pgen.1005246.s006.docx]

**S2 Table. Sequences of DNA oligonucleotides used for PCR-mediated site-directed mutagenesis of TAF_I_110 and TAF_I_68.** The sequences of DNA oligonucleotides are shown in 5’ to 3’ orientation.

| **Name** | **Primer sequence** |
| --- | --- |
| TAF_I_110/T852A | F: ACTCGCTCCCAGCAGCACGCACCCGTCC  R: AGAGCTAGAGAGGACGGGTGCGTGCTGCTGGG |
| TAF_I_68/K438R | F: CGATAGGTCCGTCGCATATAAGAGAAG  R: CTTATATGCGACGGACCTATCGATGTGTGAACGGTAG |
| TAF_I_68/K443R | F: GCATATAGGAGAAGAAAAATGGTGGAG  R: CCATTTTTCTTCTCCTATATGCGACGGACTTATCG |
